# Mobile Eye Tracking During Real-World Night Driving: A Selective Review of Findings and Recommendations for Future Research

**DOI:** 10.16910/jemr.10.2.1

**Published:** 2017-03-15

**Authors:** Markus Grüner, Ulrich Ansorge

**Affiliations:** University of Vienna, Austria

**Keywords:** eye movements, attention, individual differences, gaze, driving tasks, real-world driving, night driving, mobile eye tracking

## Abstract

We exhaustively review the published research on eye movements during real-world night driving, which is an important field of research as fatal road traffic accidents at night out-number fatal accidents during the daytime. Eye tracking provides a unique window into the underlying cognitive processes. The studies were interpreted and evaluated against the back-ground of two descriptions of the driving task: Gibson and Crooks’ description of driving as the visually guided selection of a driving path through the unobstructed field of safe travel; and Endsley’s situation awareness model, highlighting the influence of drivers’ interpreta-tions and mental capacities (e.g., cognitive load, memory capacity, etc.) for successful task performance. Our review unveiled that drivers show expedient looking behavior, directed to the boundaries of the field of safe travel and other road users. Thus, the results indicated that controlled (intended) eye movements supervened, but some results could have also reflected automatic gaze attraction by salient but task-irrelevant distractors. Also, it is not entirely certain whether a wider dispersion of eye fixations during daytime driving (compared to night driving) reflected controlled and beneficial strategies, or whether it was (partly) due to distraction by stimuli unrelated to driving. We concluded by proposing a more fine-grained description of the driving task, in which the contribution of eye movements to three different subtasks is detailed. This model could help filling an existing gap in the reviewed research: Most studies did not relate eye movements to other driving performance measurements for the evaluation of real-world night driving performance.

## Introduction

In this article, we exhaustively review eye-tracking research on real-world night driving. Night driving was chosen for two reasons. First, driving a vehicle is among the most dangerous activities humans regularly perform. Globally, in 2015 alone, 1.3 million people died due to road traffic injuries [1]. Second, important in the context of the present review, driving at night is particularly dangerous. Despite similar total numbers of crashes during daytime and nighttime, fatalities per 100 million miles are disproportionately higher at night than during the day [2]. Although fatal accidents occur for a variety of different reasons, the low luminance at night impairs human vision, as we will also briefly explain below, and thus contributes to the danger of nocturnal traffic crashes [3]. Therefore, understanding the conditions for safe night driving behavior is very important. Here, we used a systematic and exhaustive review of all existing eye-tracking research on real-world night driving to understand how much can be concluded from this research about the conditions of safe night driving. 

We focused on real-world night driving, since real-world night driving occurs under the most realistic light conditions, as opposed to driving in a simulator [4], and in a three-dimensional environment. Furthermore, as the risk of accidents is only present during real-world driving, driving behavior in the real world provides the yard-stick to which conclusions from simulator research have to be compared [5]. Therefore, we refer to simulator studies of night driving only occasionally, for example, to highlight gaps in the eye-tracking research on real-world night driving. In subsequent paragraphs, you should be building a case for your study. Explain what has been found in previous research on this topic, describe what gap exists in this literature, and explain how your study will fill the gap (i.e., provide a unique study that will contribute new knowledge in the area).

Although the ultimate goal is to identify and understand the conditions that facilitate or hinder safe, successful driving, this review is restricted to gaze behavior since eye movements provide a relatively universal window into many different cognitive processes underlying driving behavior (e.g., memory, decision making, action control, etc.). Driving depends on vision [6] and eye movements indicate from which locations in the visual environment information is picked up [7]. Admittedly, the connection between the selection of visual information from a particular location and the direction of gaze is not perfect. For example, human vision is not as sensitive during saccades (i.e., the jerky jumping movements of the eyes from one location to another) as during fixations (i.e., the phases when the eyes stand relatively still) and during smooth pursuit eye movements (i.e., the eye movements that keep track of a moving object, such as an oncoming car; cf. [8]. Thus, for meaningful interpretations of eye-tracking data, it is necessary to discriminate between different types of eye movements, such as fixations and saccades. In addition, visual information is sometimes selected from the periphery of the visual field, indicating that gaze direction and covert shifts of spatial attention are not always aligned [9-11]. However, in numerous instances, gaze direction and fixation duration are valid indicators of the kind of visual information that has been selected (e.g., 7,12). Therefore, at least gaze directions during fixations and smooth pursuit eye movements are good approximations to the visual locations from which humans select information during driving.

To answer the ultimate questions concerning helpful and harmful conditions during night driving using eye tracking, a model explaining the appropriate expedient gaze behavior is necessary. In the case of gaze directions, it is important to know which areas of the visual field contain relevant information for driving and which areas contain irrelevant or distracting information. This requires a deeper analysis of the task of driving and the subsequent localization of visual information, which is relevant to the task. Therefore, in the present review we first summarize the major characteristics of the visual situation during night driving, and secondly, we analyze the task of driving. These two introductory chapters set the proper stage for the subsequent research review.

## The Visual Situation during Night Driving

As mentioned above, fatalities are higher during night driving than during daytime driving, and one contributing factor could be the low illumination at night. Low illumination is a challenge for humans, as human vision has primarily adapted to daylight illumination, and thus, the eye is more responsive to higher illumination. During the day, illumination ranges from 1,000 lux on an overcast day to about 100,000 lux at direct sunlight [13]. During the night, these values decrease considerably, ranging from 0.0001 lux at a moonless night to 1 lux at a full moon night, meaning that there is much less stimulation of the eyes at night than during the day. Luckily, the eyes can adapt to these illumination differences. About 95% of all retinal photoreceptors are rods (about 60,100,000). These rods are highly sensitive to light and respond even to single photons. This enables humans to report the presence of a single photon with above-chance accuracy [14]. Thus, rods can compensate for low illumination at night.

Yet, rods are found in the periphery of the retina, and they are largely absent at the retinal fovea. Importantly, the periphery of the retina has a low spatial acuity because retinal ganglion cells pool the visual signal across large areas [15]. In addition, rods respond indiscriminately to light of different wavelengths, and thus, rods do not allow color discrimination. In conclusion, visual adaptation to lower illumination at night comes at the cost of lower spatial acuity (or resolution) and less color vision.

In contrast, only at the fovea, single photoreceptors connect to single retinal ganglion cells, thereby enabling a high spatial resolution of vision. Therefore, high spatial resolution depends on cones (about 3,200,000), which are concentrated at the fovea [16,17]. In addition, cones are differentially sensitive to different light wave-lengths, thereby enabling color vision. However, the absolute threshold of the cone response is about 200 photons [18], which means that cones contribute to vision only if the luminance exceeds natural twilight level (about 10 lux). At twilight level, rods already get saturated. As a consequence, in well-lit environments (i.e., during photopic vision) human vision is cone-mediated and in dark environments, it is rod-mediated (i.e., scotopic vision).

There are further differences between scotopic and photopic vision that can impact night driving performance. Cones respond very fast to altering illumination [19], enabling the visual system to detect flicker exceeding 100 Hz in peripheral vision during bright light [20]. Furthermore, the highest contrast sensitivity of the cones at the fovea (i.e., a detection of 0.5% spatial or temporal contrast) is found under photopic light conditions. Under scotopic light conditions, contrast sensitivity of the cones is much lower (more than 5% contrast is needed). The actual sensitivity of the human visual system also depends on the light adaptation of the retina. Here, cones recover their photocurrent much quicker (within 20 ms after full bleaching) than rods (which take 20 min to recover their full contrast sensitivity; ,21). Therefore, in addition to the lower color sensitivity and the decreased spatial acuity, the lower sensitivity to temporal flicker and spatial contrast and the longer duration to recover full spatial contrast sensitivity are further disadvantages of scotopic vision that potentially contribute to decreased uptake of visual information at night, and hence to impaired driving performance during night driving compared to daytime driving.

To compensate for the lack of natural light at night, car headlamps and road lighting are used to illuminate the driving scene. The headlamps are especially important for drivers to detect and recognize unlit objects and hazards at night, for example, animals or pedestrians ([22-24]. Nevertheless, daylight illumination is never achieved. The illumination provided by headlamps falls mainly into the range of twilight illumination, meaning that there is enough light for cones and not too much light to saturate the rods so that both photoreceptors contribute to what is called mesopic vision [25]. During mesopic vision, complex cone and rod interactions may occur [26], for a review, see Stockman and Sharpe [27].

In addition to the low illumination, visual glare challenges drivers’ vision during night driving, and these factors interact with non-visual factors, such as drivers’ age or (age-dependent) visual impairments. For example, glare and light reflections impair object and hazard detection and recognition particularly in older drivers [28-30] However, also non-visual factors like alcohol consumption and fatigue that often coincide with night driving contribute to the risk of fatal crashes [2].

## The Task of Driving

Although lower illumination at night provides challenging visual conditions to human drivers, humans are usually capable of relatively safe night driving. One trivial reason is that ambient light, headlamps, and lighted or reflective objects ensure that relevant visual information can easily be seen even during night driving. However, more interestingly, not all task-relevant visual information is equally affected by nocturnal light conditions. Object detection and recognition that depend on visual acuity at the center of the visual field are more degraded by the low illumination during night driving than lane and distance keeping, which do not depend on high visual acuity [31-33]. Thus, in many but not all respects driving performance is relatively robust against the visual challenges of night driving [34] and only an in-depth analysis of the task of driving will allow us to understand which visual information is task-relevant and needs to be recognized by the driver.

**Figure 1:**
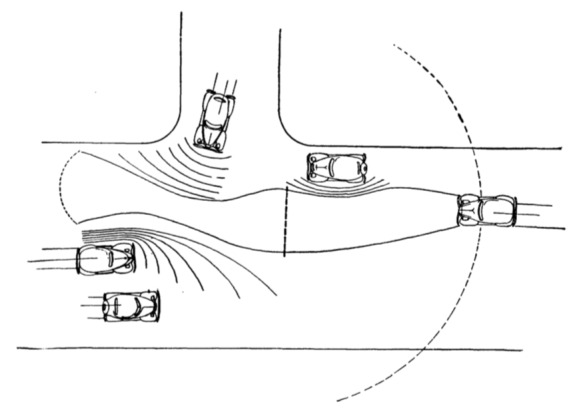
A sketch of the field of safe travel for the car on the right. The vertical line in the center of the field of safe travel marks the minimal stopping zone. From American Journal of Psychology. Copyright 1938 by the Board of Trustees of the University of Illinois. Used with permission of the University of Illinois Press.

A historically early, straightforward formal description of the task of driving was provided by Gibson and Crooks [35]. These authors based their description of the driving task on a perceptually defined field of safe travel. In this task description, driving is conceptualized as locomotion through a field of space, and the field of safe travel corresponds to the part of the space through which locomotion is safe. The main purpose of locomotion is to move safely from one place to another. This is achieved by avoiding obstacles and other hazards based on visual information in the environment. The necessary adjustments of locomotion are considered to correspond to a visually guided path selection through the field of safe travel [35]. During driving, the field of safe travel would consist “of the field of possible paths which the car may take unimpeded” [35]. Therefore, its boundaries are obstacles (e.g., the shoulder of the road) which (potentially) impede safe travel. Such obstacles have a negative perceptual valence in a driving task, whereas the field of safe travel has a positive valence, especially its center (viewed from the perspective of the driver) where the boundaries are the farthest away. Furthermore, the model incorporates a minimal stopping zone, which is the area in front of the car through which the car would have to pass before it could finally be brought to a halt in case of an emergency stop. Figure 1 depicts an exemplary field of safe travel including the minimal stopping zone.

For safe driving, the minimal stopping zone should be within the larger field of safe travel, and a larger field-to-zone ratio should be preferred over a lower one. Importantly, the description defines the boundaries of the field of safe travel not only by physical obstacles but also by other situational circumstances, such as intersections, speed limits, traffic rules, including areas where obstacles could potentially appear. For example, the edge of the headlamp beam during night driving would be such an area. During night driving, new obstacles would become visible first when crossing the edge of the headlamp beam, and therefore, drivers should carefully monitor this area.

However, it is questionable whether the task description of Gibson and Crooks [35] is general enough to do justice to all situations that come up during driving. For example, at times, active search for particular information, such as for traffic signs indicating allowed maximal speed, could supersede the search for the boundaries of the field of safe travel. Such driving tasks be better conceptualized within general decision models, like the situation awareness model from Endley [36]. Situation awareness is based on a complete assessment and accurate comprehension of the situation, but this requires more than the processing of incoming visual information. Situation awareness is an understanding of the meaning of the visual information and how it relates to the goals of the person in a specific situation or task. Thus, it can be adjusted to cover a wider range of driving tasks.

The situation awareness model is more sophisticated than the model of Gibson and Crooks [35], as it includes not only environmental or visual but also organismic factors, such as the driver’s attention, goals, working memory, cognitive load, perceived complexity of the situation, automatization of actions, and mental models. The latter denote flexible semantic structures of drivers that would allow them to represent even theories, for example, about future events. This description of the task of driving highlights some factors that are also important for safe driving but were not reflected upon in the earlier model of Gibson and Crooks [35].

Endsley [36] defined situation awareness as a state of knowledge, dependent on but at the same time separate from the processes needed to acquire this state, which she labeled situation assessment. Additionally, Endsley [36] distinguished situation awareness from decision making and performance itself. Even with perfect situation awareness, drivers might make wrong decisions or show poor performance, but with inaccurate situation awareness, even perfect decision makers and performers would go wrong. Other important factors affecting situation awareness are attention and working memory, which in turn are influenced by the current workload, stress, and the perceived complexity of the situation.

Situation awareness consists of three hierarchical levels. At the first level, the person perceives elements of the environment. For example, a driver perceives obstacles, her own speed, etc. At the second level, a driver comprehends the current situation by understanding the significance of the perceived elements. For example, she understands whether other road users constitute a hazard or not. The second level is situation comprehension. It integrates the relation between the elements as well as the elements’ locations in the environment. Due to this difference between first and second level processing, inexperienced drivers might perceive the same elements as experienced ones but might fail to comprehend the situation appropriately if they do not understand the significance of the relations or the locations of the perceived elements for their goals. The first and second levels together enable the third and highest level of situation awareness. Understanding the significance of the perceived elements allows the person to anticipate the future of the situation and, therefore, to act more effectively and to make better decisions.

With the help of the situation awareness model of Endsley [36], night driving can be defined as a special case of driving in which it is more difficult for the driver to accomplish a high level of accurate situation awareness because of the decreased visibility of the environment at night. Furthermore, some persons are more affected than others by nocturnal light conditions. Thus, organismic factors might contribute to impaired situation awareness during night driving.

Related to the idea of sorting visual information into task-relevant and irrelevant information on the basis of task definitions, it is possible to discriminate between controlled and automatic forms of visual attention and eye movements [37]. In Trick et al.’s [37] framework, visual attention corresponds to the selection of visual information from the environment, a process tightly linked to eye movements (7, see also above). In this conception, the controlled forms of visual attention (being slow, effortful, intended, and more flexible) would correspond to the selection of task-relevant visual information. In contrast, automatic forms of visual attention (being fast, effortless, and unintended) could correspond to the distraction caused by task-irrelevant information, too. By adding a second, orthogonal dimension (origin of the selection principle: exogenous or stimulus-driven vs. endogenous or driven by prior experience and mental representations) to their attention framework, Trick et al. [37] discerned altogether four different types of visual attention: (a) reflexes (automatic-exogenous), (b) habits (automatic-endogenous), (c) exploration (controlled-exogenous), and (d) deliberation (controlled-endogenous).

## The Reviewed Studies

After having described the visual situation at night and the task of driving, we next review existing eye-tracking research on real-world night driving. However, the studies reviewed used different theoretical rationales and sometimes lacked a clear theoretical rationale altogether. Therefore, it was not possible to summarize these studies with direct reference to any of the task-related factors discussed above. Instead, we categorized the reviewed studies by two orthogonal dimensions that most closely resembled important characteristics of the above models. One dimension that we used to categorize the reviewed studies was the environmental versus organismic origin of the factors modulating behavior. We asked if a factor that modulated eye movements during real-world night driving corresponded to a factor located in the environment (e.g., the headlamp condition used, the road geometry, the presence vs. absence of traffic signs, etc.) or whether it corresponded to a factor located within the driver’s organism (e.g., the driver’s age, her experience, etc.). This dimension connects to the exogenous-endogenous distinction of Trick et al. [37] and also allowed categorizing many of the influences listed in the situation awareness model of Endsley [36]. For example, we would have categorized Endsley’s elements of a situation, such as traffic signs or obstacles, as environmental factors, whereas we would have categorized task-relevant factors, such as cognitive load, as organismic.

The second dimension that we used to categorize the reviewed studies was the temporal inertia with which a factor exerted its influence. Some factors would have exerted a uniform influence for more extended durations – that is; the influences would have typically remained unchanged for minutes, hours, days or sometimes even years. Examples for such more stable or inert influences would be the weather conditions, the drivers driving experience or the age of the driver. Some automatic influences à la Trick et al. [37], such as a learned habitual tendency to look for traffic signs at roadside locations, would correspond to these stable factors. The influences of other factors could have varied more drastically in a relatively short time – that is, from moment to moment. For example, cognitive load by a demanding secondary task could have increased momentarily when a situation would have required driving and at the same time scanning the roadside for potential hazards. Table 1 provides an overview of different factors and how they roughly corresponded to the poles of the two dimensions of our categories.

**Table 1:** The framework used to structure the results of the reviewed literature.

	Environmental Factors	Organismic Factors
Stable	· Road geometry (curves, straight sections)	· Visual capacity
	· General light condition (night/day, unlit/lit street, headlamp)	· Mental capacity
	· Road type (number of lanes, pavement markings)	· Personality
	· Road environment (city, suburban, rural, highway)	· Driving experience
	· Weather condition (dry, wet, rain, fog, snow)	· Familiarity with road and car
		
Variable	· Other road users (oncoming and preceding traffic, pedestrians)	· Cognitive load
	· Signs and signaling devices (traffic lights, delineation treatment)	· Fatigue
	· Object and hazards	· Alertness
	· Glare	· Drug influence
	· Traffic situation (intersections, overtaking)	
	· Interaction with car (assistance systems)	
	· Light-induced dynamics caused by adaptive headlamps	

## Method

Focusing only on research that measured eye movements during real-world night driving enabled us to review view all published literature exhaustively. We used a combination of the keywords “night” or “dark” or “darkness”, and “driving”, and “eye movements” or “eye tracking” to search in the databases Web of Science, PsycARTICLES, PsycINFO, TRID (includes Transportation Research Information Services and International Transport Research Documentation), and the Transportation Research Board Publication Index. Additionally, we extended our search to Deep Blue, the institutional repository of the University of Michigan (http://deepblue.lib.umich.edu), and the search engines Google Scholar, Google, and Bing.

In the next step, we scanned the results for studies that used a real-world setting and measured drivers' eye movements. In the papers that met this requirement, we looked for new references that we missed earlier. The final list contained 29 publications (see Table 2). We excluded studies that used eye tracking to measure the effectiveness of an algorithm [38] or very specific traffic interventions [39], such as reflective pavement markers [40] or ground mounted diagrammatic entrance ramp approach signs [41,42]. Furthermore, we excluded [43], because they investigated the gaze distribution related to the windshield without considering the traffic situation, and [44], who did not report the analysis of his real-world eye tracking data.

**Table 2:** All reviewed publications which measured eye movements during real-world night driving.

Rockwell, Ernst, and Rulon, 1970 [46]
Mortimer and Jorgeson, 1974 [49]
Mortimer and Jorgeson, 1974 [50]
Rackoff, 1974 [55]
Rackoff and Rockwell, 1975 [56]
Graf and Krebs, 1976 [45]
Mourant and Mourant, 1979 [57]
Zwahlen, 1981 [58]
Zwahlen, 1982 [47]
Zwahlen, 1987 [59]
Zwahlen, 1988 [60]
Olson, Battle, and Aoki, 1989 [61]
Zwahlen, 1993 [62]
Zwahlen, 1995 [63]
Zwahlen and Schnell, 1997 [64]
Schnell and Zwahlen, 1999 [65]
Zwahlen, Russ, Roth, and Schnell, 2003 [41]
Schieber, Burns, Myers, Willan, and Gilland, 2004 [66]
L. L. Higgins, Ko, and Chrysler, 2009 [67]
Ko, Higgins, Chrysler, and Lord, 2009 [54]
Carlson et al., 2010 [68]
Dukic, Ahlstrom, Patten, Kettwich, and Kircher, 2013 [69]
Brimley, Carlson, and Hawkins, 2014 [48]
Cengiz et al., 2014 [51]
Maxera, Kledus, and Semela, 2015 [70]
Theiss, Swindell, Gillette, and Ullman, 2015 [71]
Theiss, Gillette, and Ullman, 2015 [72]
Winter, Fotios, and Völker, 2016 [52]
Hartmann, Grüner, Ansorge, Büsel, and Bednar, 2016 [73]

## Results

Our review is organized along the dimensions of our categories, starting with the stable environmental factors. In each section, we first quickly review the major findings concerning different factors in turn and then evaluate them against the background of driving models that we have presented in the Introduction. Only this evaluation allowed us to interpret what the findings from the eye-tracking studies might have meant regarding the cognitive processes involved and their potential benefits and harms for successful night driving.

### Stable environmental factors

#### Effects of illumination on eye movements.

During night driving, the headlamps are the most important source of light and heavily influence what the driver perceives. General differences in gaze behavior between daytime and night driving are therefore at least partially influenced by stable illumination differences. For example, during night driving, the fixations of the drivers are concentrated nearer to the own car while during daytime driving fixations are concentrated farther in the distance [45-47]. Related to this, fixations are somewhat confined to the illuminated area during night driving but more broadly distributed during daytime driving [48]. Perhaps also related to these observations, mean fixation durations tended to be longer during night driving [47, 49, 50].

More recently, Cengiz et al. [51] investigated the fixation distributions of three drivers during night driving along rural roads. The two inexperienced drivers’ fixations were concentrated farther ahead of the own car during daytime driving and nearer to the own car during night driving. During road sections illuminated by street lighting, fixations were shifted slightly farther ahead compared to driving during unlit road sections. This finding indicates that illumination by street lighting elicited gaze behavior more similar to that during daytime driving. Only the vertical fixation dispersion was higher during night driving compared to daytime driving, and interestingly, the fixation distribution of the experienced driver was the same during daytime and night driving [51].

Mortimer and Jorgeson [49, 50] investigated the effects of different headlight patterns on fixations. The headlights that were used illuminated the right side of the road more than the left side. As a consequence, during night driving, 6% of all fixations were located on the right roadside. This compares to only 2.5% during day-time driving. Graf and Krebs [45] also found slight differences between different headlights, but they found no systematic influences on eye movements. Although there is evidence that the headlight pattern and the illumination both influence eye movements, the results of Cengiz et al. [51] showed that the highest density of fixations did not coincide with the highest illuminated area. It seems that the distribution of fixations is not strictly correlated with the illumination of an area.

#### Evaluation of illumination effects on eye movements. 

Assuming that sampling by fixations from a broader distribution of locations during daytime driving, especially fixating at regions farther ahead in driving direction, allows the driver to assess the boundaries of the field of safe travel (cf. [35]), the more spatially refined fixation behavior, especially the fact that fixations were distributed closer to the driver’s own vehicle, indicated that headlight-elicited illumination during night driving was suboptimal for driving performance. In addition, it stands to reason that a more widespread distribution of fixations on illuminated locations during daytime driving would also allow the driver to recognize potential hazards and obstacles at an earlier point in time. Luminance would thus also allow better planning ahead of driving performance in general – that is, it would increase situation awareness.

However, it is conceivable that at least some fixations during daytime driving reflected distractions by elements at the roadside. The number of such fixations on task-irrelevant distractors has not been assessed in the aforementioned studies, but this would be necessary for a sound conclusion. In addition, most of the general influences of nocturnal light conditions on fixations should be treated with caution because many other factors could have interacted with illumination, for example, the road environment [52], the driving experience [53], and specific traffic situations [54]. These influences will be covered next.

#### Road geometry and road type.

The course of the road influences mainly the spatial distribution of eye movements, probably because it is directly related to the driver’s task of lane keeping. Accordingly, right and left curves elicit different fixation distributions. During the negotiation of left curves, fixations are concentrated on the left roadside or the center line markings (the left side of the driver’s own lane), while during right curves more fixations are directed to the right roadside, and in straight sections, fixations are concentrated mostly at the center of the road [45, 49, 50]. These general gaze patterns are found during night and daytime driving, which indicates that the effect of road geometry is independent of illumination. Although the distribution of fixations is heavily influenced by the road geometry, [62] found no systematic fixation sequence patterns of drivers while driving through right curves.

Pavement markings are stable features of specific road types which also influence eye movements – if they are visible. For example, the center line markings in the study of Mortimer and Jorgeson [49, 50] were considerably less likely fixated during night driving than during daytime driving, presumably indicating that the lines were difficult to see with the headlamps used.

However, if pavement markings are bright and highly visible, fixations tend to concentrate farther in the distance, potentially indicating that easily recognizable pavement markings facilitate peripheral recognition of the lane, such that fixations can be directed farther ahead of the own car since there is less need to look at markings in the near distance [64,65]. Furthermore, fixations are generally more concentrated when the pavement markings are bright and easily visible compared to a wider fixation distribution when pavement markings are harder to see [74].

#### Evaluation of the influences of road geometry and road type.

Assuming that fixations on pavement markings for lane keeping and fixations ahead of the car for assessment of the field of safe travel are competing for a limited processing capacity, the fact that easily recognizable pavement markings elicited more fixations in the distance than less visible markings might have reflected a lower cognitive load in the easier perceptual task (cf. [36]). This implies that well visible road markings would enhance driving performance. The results also suggest that controlled and automatic influences on attention should be orchestrated. Assuming that a high visual salience of the markings allowed their automatic recognition in peripheral vision (e.g., maybe even without a shift of visual attention or without fixations on these positions), more capacity for controlled shifts of spatial attention to other task-relevant areas in driving direction were possibly set free (cf. [37]).

#### Road environment.

Winter et al. [52] investigated the fixation distributions during night driving down a main traffic road and through a residential area. They found that on the main traffic road the area in which 95% of all fixations occurred was elliptically shaped, but the areas in which 25% and 50% of the fixations occurred were circular (all these areas were straight ahead of the driver). In contrast, when driving through a residential area, fixations of all frequency ranges were located in elliptically shaped areas [52]. This finding indicates that fixation distributions depend on road environments, with higher horizontal dispersion when driving through a residential area compared to a main traffic road. Furthermore, when driving through a residential area, the fixation distribution was shifted to the right of the driver’s own lane, while on the main road fixations were concentrated at the center of the driver’s own lane [52].

#### Evaluation of the influence of road environment.

The results seem to echo those found with more or less visible markings reviewed above if one assumes that the driver expected more potentially relevant information at the roadside in residential areas than on main traffic roads. For example, the driver’s expectation of careless pedestrians crossing the street or playing children entering the road from the side could be reasonably higher in residential areas, where traffic is weaker compared to main roads. This might have prompted the driver’s adaptive monitoring of the roadsides when driving through residential areas. The fact that more eye movements were directed to the right roadside would be in line with this interpretation because with right-hand traffic, as was investigated here, pedestrians crossing the street from the right roadside would have to be noticed earlier by the driver to avoid accidents compared to when participants cross the street from the left roadside. However, this conclusion comes with the same uncertainty as for the one concerning the comparison of daytime and night driving that we analyzed above. Unless the researchers would have determined if the eye movements to the roadsides were directed at potentially relevant information or driving-unrelated distractors, it is difficult to assess if the gaze behavior is functional. The minimum measure that would be recommended for such studies would be to ask the drivers for their intentions in these situations (e.g., to ask them whether they actively monitored the roadsides for street crossings on residential and on main roads).

#### Weather conditions.

To date, there are no published studies about the influence of adverse weather conditions (rain, fog, snow, etc.) on eye movements during night driving. However, a driving simulator study indicated that rain during day-time driving affected eye movements similarly to night driving – that is, less and longer fixations were observed in the rain condition [75]. Adverse weather conditions increase the risk of accidents [76], and more studies (including real-world night driving studies) are therefore desirable.

### Variable environmental factors

#### Traffic situation (oncoming traffic, glare, intersections, and preceding cars).

Mortimer and Jorgeson [49,50] investigated the influence of oncoming traffic on eye movements during daytime and night driving. Generally, they found that drivers reduced their fixations on locations inside of the car when oncoming traffic was present, and the number of fixations on the oncoming car increased as it drew closer. This indicates that drivers paid attention to the situation on the street, and an on-coming car is perceived as an important object.

The task of keeping an eye on oncoming traffic also seems to override the influence of headlamp light patterns on eye movements. When the headlamp light pattern would have shifted the fixation distribution of drivers to the right roadside, as reported by Mortimer and Jorgeson [49,50], once oncoming traffic was present, the number of fixations on the right roadside was reduced to 2.5%, which was very similar to daytime driving with and without oncoming traffic. This once again highlights the importance of oncoming traffic for the driver and shows that situational requirements override the effects of headlamp illumination on eye movements.

During night driving, oncoming vehicles were fixated even more frequently than during daytime driving [49,50]. This might have been the case because the bright headlamps of an oncoming car are more salient at night than during the day. However, these fixations might also serve recognition. The glare of oncoming cars caused a shift of the fixation distribution towards the lane of the oncoming car [45], which indicates that the glare impaired the detection of objects at the roadside. Unfortunately, Graf and Krebs [45] did not investigate in any detail object detection when a glaring car passed. They only reported “[...] in a few cases, a target fixation” (p. 56) and a very short (about 15 m [50 ft] or less) detection distance. Other studies showed that glare impairs recognition of pedestrians [77] – an important finding, as the adverse effect of glare is often underestimated [22].

In situations with a preceding car, drivers’ fixations tend to be more focused on the preceding car, especially during night driving compared to daytime driving and to driving without a preceding car [61] Importantly, Olson et al. [61] did not instruct the drivers to follow the preceding car, which would have most probably increased the number of fixations on the preceding car [78]. That fixations on preceding cars were found nonetheless makes sense as the preceding car is highly relevant for speed adjustments and avoidance of a rear-end collision.

L. L. Higgins, Ko, and Chrysler [67] and Ko et al. [54] investigated eye movements at intersections during night driving and found that the frequency of fixations on the left side increased and that the frequency of fixations on the right side decreased when drivers turned left at intersections. When turning right or driving straight through the intersection, fixations were more frequent on the ride side and straight ahead. These effects became more prevalent as the drivers approached the intersection [54,67].

#### Evaluation of the influence of traffic situations.

The findings in this area indicated that participants conducted controlled eye movements (cf. [37]) that were in line with the requirements of the driving task. In support of a correct interpretation of the driving situation and high situation awareness (cf. [36]), preceding and oncoming cars and road conditions in the heading direction at intersections were all fixated, probably because potential limitations of the field of safe travel [35] at these locations were anticipated. However, with the exception of the gaze behavior at intersections, many of the corresponding eye movements might have also reflected automatic influences of stimulus salience (cf. [37]). High local luminance contrasts created by the headlamps of oncoming traffic and by the tail lights of preceding cars during night driving as well as the dynamics of the moving cars during daytime and night driving could have attracted the gaze in an automatic way, by means of stimulus salience, too (cf. [79]). But generally, more evidence favors a looking strategy, which is appropriate for specific driving tasks. The gaze behavior at intersections was clearly not dominated by stimulus salience alone but more likely driven by the requirements of the driving task. However, this does not mean that salience is insignificant. In fact, salience could be a powerful automatic attractor of the gaze and task-relevant objects (e.g., cars) should be equipped with salient features (e.g., head-lamps).

#### Salience of objects, signs, and pedestrians

Graf and Krebs [45] investigated the detection of objects which might occur naturally along a road (e.g., a small carton, a car muffler, a rabbit skin, a pedestrian model). In one condition, drivers did not know that there were objects at the roadside. In this non-alerted condition, objects were detected earlier during daytime driving compared to night driving. Interestingly, there was no significant difference between different headlamps. Graf and Krebs [45] replicated this result in their second experiment, in which non-alerted drivers detected the object only slightly earlier when driving with high beam compared to low beam. This suggests that the better illumination provided by high beams does not necessarily lead to better target detection when the drivers are not instructed to search for specific objects. Also notable was the high variability between participants concerning the largest distances that allowed successful object detection: This indicated that organismic factors (e.g., visual acuity) could have played a considerable role in object detection during night driving.

Generally, drivers detect more salient objects earlier than less salient ones [68]. The salience of objects depends on features of the object itself. For example, bigger or stronger illuminated objects appear more salient and tend to attract more and longer fixations compared to smaller and less illuminated (i.e., less salient) objects [69]. Especially pedestrians are often difficult to see at night [24], and therefore, should be salient or conspicuous, even more so as pedestrians tend to overestimate their visibility at night [23]. Maxera, Kledus, and Semela [70] measured pedestrian detection using eye tracking and found that during night driving, drivers spotted pedestrians at a pedestrian crossing relatively late. In such situations, increasing the salience of pedestrians proved effective for enhancing earlier detection of pedestrians [23]. However, comprehensive studies investigating the connection between eye movements and pedestrian detection during night driving in real-world settings are missing.

Like salient objects, salient light-induced dynamics caused by an adaptive headlight beam could attract the gaze and elicit eye movement changes. Such adaptive headlamps provide illumination similar to high beams, while at the same time avoid glaring other road users by adjusting the driving beam [80]. This adjustment introduces a new kind of light dynamic to the driving scene – a dynamic that is not a mere consequence of the driver’s own car movement, but that also depends on the movements of the oncoming traffic and the road geometry. In a pilot study, Hartmann, Grüner, Ansorge, Büsel, and Bednar [73] showed that such light-induced dynamics of the driver’s own headlamp attracted eye movements during real-world night driving.

Zwahlen [58-60] investigated the specific effects of salient traffic signs on eye movements and found that drivers generally fixated signs two times before they passed them and that the fixations took 0.5 to 0.85 s. During both daytime and night driving, drivers’ first fixations on a sign occurred about 7 s before they passed the sign [58].

Dukic et al. [69] investigated the effects of salient electronic billboards on eye movements during daytime and night driving. Drivers fixated electronic billboards more often and longer compared to other traffic signs, probably because the electronic billboard signs were lit and therefore brighter than the only retroreflective traffic signs. The billboards were also bigger than the traffic signs, and, to some extent, they were also more dynamic (changing their messages every 7 s) than the traffic signs. Dukic et al. [69] found no differences between daytime driving and night driving, although at night the billboards appeared brighter than during the daytime. This finding indicated that salience alone might not have been the only influence in these situations. For example, more information content on the billboards could have also invited more explorative fixations on the billboards than on the traffic signs.

Theiss, Swindell, Gillette, and Ullman [71] and Theiss, Gillette, and Ullman [72] investigated eye movements during daytime and night driving through road construction zones. Eye movements were measured while the participants made turns into correct gaps between channelizing devices. The channelizing device which produced the lowest error rate (i.e., the least missed turns) was easily detectable and elicited with more and longer fixations [71,72]. Since the participants had to recognize the driveway indicated by the channelizing devices, longer and more fixations on the channelizing devices might have improved performance. However, in other situations, more and longer fixations on driving-unrelated objects might be detrimental to driving performance (e.g., 69).

Schieber et al., [66] investigated the reading of highway signs at night using fixations to measure reading distances. Their participants knew what the targets looked like and had to report the detection of the target signs verbally. Higher sign luminance tended to elicit longer fixations, with average fixation duration above 3 s. These long fixation durations were contrary to the results of other studies, which indicated fixation durations around 0.5 s [59]. It is possible that the task of verbally reporting the message of the sign prolonged fixation durations because the drivers wanted to be sure that they read the sign correctly.

#### Evaluation of salience of objects, signs, and pedestrians.

We summarized the influence of visual salience under the variable factors, although in general, saliency affects eye movements probably in an automatic way and a wide variety of different situations [79, 81, 82]. However, the reader should note that by variability we here referred to the inertia with which changes in salience can alter eye movements and this inertia is seemingly very low for salience [83,84] Salience, such as a high local feature contrast in terms of luminance, color, or orientation [79], is an extremely powerful and automatic attractor of attention and eye movements (cf. [37]). This was also found throughout the night driving studies that we reviewed here. Whether salience helps or hampers driving performance is a matter of which objects carry a high salience. In the reviewed examples, we have seen that a highly salient channelizing device signaling the lane is helpful to find the lane, but we have also seen that salient task-irrelevant distractors, such as billboards, have the potential to distract the gaze away from probably more relevant locations. However, the latter example also made clear that information content rather than salience alone could be more decisive for gaze distraction: Although the billboards had a higher luminance contrast relative to their dark surroundings at night than during daytime, the gaze was equally attracted by the billboards during daytime and night driving. In conclusion, the finding that salience or information content can distract the gaze makes it once more clear that the larger dispersion of the fixation positions during daytime compared to night driving might not only reflect task-relevant fixations during driving.

### Stable organismic factors

#### Driving experience.

Although there is quite some literature concerning the influence of experience on driving [53,75,85–91], the eye movements of experienced and inexperienced drivers during real-world night driving have not yet been investigated. Nevertheless, there is one small clue to the influence of experience: The fixation distribution of the most experienced driver (200,000–300,000 km driving experience compared to 10,000–30,000 km of the other two drivers) tested by Cengiz et al. [51] was very similar during daytime and night driving. Since only one experienced driver was tested, this result could very well be just an exception. However, perhaps experienced drivers have learned where to look at in a specific driving situation, and different light conditions hardly influenced this learned gaze behavior. Future research should compare the gaze behavior of experienced and inexperienced drivers during real-world night driving to test this idea.

#### Visual and mental capacity (age).

The influence of (impaired) visual and mental capacity is often investigated by comparing young and aged drivers because old age often involves a decline in visual and mental capacities [28,29,55,92,93]. These impairments are especially harmful during night driving, but not all aged drivers are affected to the same extent. For example, Rackoff [55] and Mourant and Mourant [57] found essentially no difference between younger and aged drivers regarding fixation distributions and durations. Schieber et al., [66] found no influence of age on the maximally allowed distance for successful sign reading. Maybe this was due to a self-selection effect. It could be that only aged drivers without visual and mental impairments volunteered in these studies and therefore no or very few differences were found. Indeed, older drivers that experience visual and cognitive impairments tend to avoid challenging driving situations, like night driving or driving in adverse weather conditions [94].

Importantly, impaired vision caused by old age or nocturnal light conditions (or both) affected not all driving tasks equally. For example, lane keeping and speed control were largely unimpaired, probably because these tasks can be accomplished with the help of blurry peripheral vision, too. However, object recognition, for example, depends on good visual acuity and is severely impaired at low illumination or impaired visual acuity [32,33,24] see [95] for a detailed review about driving and vision).

Gibbons, Edwards, Bhagavathula, Carlson, and Owns [96] found that age significantly influenced object detection: younger participants outperformed older ones. Furthermore, higher ambient illumination and object luminance facilitated object detection. The same factor influenced pedestrian recognition as well: More salient pedestrians were recognized earlier, while age impaired pedestrian recognition [30,97].

#### Evaluation of the influence of visual and mental capacity (age).

Clearly, age-related decrements of vision and mental capacity have the potential to diminish night driving performance. Also, there are indications that some driving-related performances, such as lane keeping could be spared. This is reminiscent of the findings regarding fewer fixations on well visible compared to less visible road markings that we reviewed above. The dissociations between peripheral and central vision suggest that it could be sensible to specify further subtasks, an issue that we will pick up in the General Discussion. However, the currently reviewed findings of age-related influences have to be treated with caution. Nowhere in the studies that we have reviewed so far is a self-selection bias as likely as in aged drivers: Aged drivers would rather not participate in real-world driving studies if they feel that their visual and mental capacity has already declined. Thus, what has been reported in the published studies could well be an underestimation of the true driving performance drop that comes with age.

#### Personality.

Since the observation of Greenwood and Woods [98] that only a small proportion of individuals were involved in most of the accidents, other studies also indicated that some individuals are generally more prone to accidents than others [99,100]. But the concept of accident proneness as a personality trait remains elusive [101]. There are no studies connecting eye movements during night driving with accident proneness.

### Variable organismic factors

#### Alertness.

The influence of alertness on eye movements during real-world night driving was only addressed once, by Graf and Krebs [45]. They compared non-alerted drivers, who were unaware that objects were presented along the roadside, semi-alerted drivers, who were instructed to call out detected objects, and fully alerted drivers, who were informed in advance which objects to look for. The results in the respective conditions were quite different. Non-alerted drivers’ fixations were centered at the middle of the road, whereas alerted drivers’ fixations were more frequent at the right roadside, where the known objects would appear, and semi-alerted drivers’ fixations were broadly distributed along the horizontal axis, probably representing search behavior for unknown objects. During the fully alert condition, drivers hardly looked at the road anymore, which indicates that searching for a known object while driving corresponds to a different task than natural driving: As the alerted drivers mainly searched for the objects instead of driving safely, these drivers detected the targets earlier than the semi-alerted and the non-alerted drivers. But the semi-alerted drivers were only slightly better than the non-alerted drivers. This result demonstrates the influence of tasks on eye movements.

#### Evaluation of the influence of alertness.

The influence of alertness nicely illustrated how important it can be to determine the task of a driver before evaluating her gaze behavior. In the study of Graf and Krebs [45] drivers in different alerting conditions were subjected to single- versus dual-task conditions, respectively, again indicating that cognitive load had an impact on task performance (cf. [36]). Only drivers in the fully alert condition drove under a dual-task condition because they drove (Task 1) and searched for objects (Task 2) at the same time. In contrast, drivers in the non-alerted condition drove under a single-task condition because they were not instructed to also search for objects (and this search behavior was also not shown spontaneously) so that they only performed one task: driving. The higher cognitive load of the fully alerted drivers was probably detrimental to their driving performance. Gibson and Crooks’ [35] field of safe travel model suggested that functional eye movements for the task of driving are directed at locations straight ahead of the driver. However, such eye movements were almost absent in the full-alerted condition. In contrast, the non-alerted group showed exactly the predicted gaze behavior, which is among the reviewed studies by far the strongest evidence for the utility of the straight-ahead fixations during driving. As such, the experiment of Graf and Krebs [45] also illustrated the strong influence of controlled processes during driving (cf. [37]).

#### Fatigue.

Fatigue is an important factor during night driving which is often not considered but highly prevalent during night driving [102,103]. Hallvig et al. [103] showed that lane departures, eye-blinking durations, and subjective ratings of sleepiness all increased during night driving. To our knowledge, no published studies investigated the influence of fatigue on eye movements during real-world night driving. This is quite surprising since fatigued drivers are disproportionally often involved in accidents because the driving performance generally deteriorates with increased fatigue [104,105].

#### Evaluation of the influence of fatigue.

Fatigue is definitely a dangerous condition and leads to many traffic accidents. It is not explicitly covered in any of the driving models that we have reviewed in the Introduction, but fatigue diminishes the ability to concentrate and to perform successfully on cognitive tasks. Thus, its influences would be covered by the situation awareness model of Endsley [36].

#### Drugs

Drugs like alcohol contribute to the accident rate at night as well [2,106]. For understandable reasons, there seem to be no studies about the influence of drugs on eye movements during real-world night driving, but a simulator study showed that alcohol consumption influenced eye movements whereas marijuana consumption did not [107]. Even small doses of alcohol can have detrimental effects on driving performance, especially at nocturnal light conditions [108], but the effects are complex. For example, alcohol might interact with fatigue or increase risk taking [109], which means that the effect of alcohol or drug consumption could be relatively indirect.

## General Discussion

Our review has shown that despite a lack of reference to a coherent description of the driving task in the studies reviewed, the findings can be interpreted in light of such descriptions. This was true for both driving models that we discussed in the Introduction: Gibson and Crooks’ [35] description of the driver’s task and the situation awareness model of Endsley [36]. For example, in line with Gibson and Crooks’ assumption of the importance of the boundaries of the field of safe travel, participants indeed spent much of their time looking at locations ahead of their cars in the direction of movement (e.g., [51]). During intersections, the drivers also spent more time looking at locations lying in the direction of their planned movements [67], a behavior that reflected the increased importance of the future boundaries of the field of safe travel. A shortcoming of the model of Gibson and Crooks [35] is its focus on visual signals alone. Even if a visual signal is seen, drivers must correctly interpret it for successful task performance. This was only acknowledged as important in Endsley’s situation awareness model. With its explicit reference to organismic factors of individuals (drivers), such as cognitive load, attention, and working memory, the situation awareness model does account for interindividual variability in task performance. This model was supported both by the finding that the performance on two simultaneous tasks – and thus, under overall increased cognitive load – diminished eye movements functional for safe driving [45] and by the general influence of organismic factors, such as drivers’ experience or drivers’ age, on gaze behavior (e.g., [96]).

However, sometimes a lack of proper control conditions left us uncertain about how to interpret the eye movements of the drivers. This was particularly true for the greater dispersion of eye movements during daytime compared to night driving [45-47] and during driving down roads in residential areas compared to driving down main traffic roads [52]. As long as the corresponding eye movements are not related to other task performance measures – but see Graf und Krebs [45] for an exception –, such as lane keeping accuracy, speed, or distance keeping to preceding traffic, it is difficult to assess whether the greater dispersion of fixations during the day was beneficial or detrimental to driving performance. Therefore, future eye-tracking studies of real-world night driving should incorporate task performance measures additionally to eye movements. At least, questionnaires to identify the intentions of the drivers should be included. This would also help to understand which gaze behavior was driven by goals and intentions and which was automatic and stimulus-driven alone. This distinction has been highlighted in the attention framework of Trick et al. [37] in which controlled (intended) and automatic causes of attention shifts and eye movements were distinguished. For example, in many studies it was not only unclear whether a factor’s influence on eye movements reflected beneficial or harmful effects on overall driving performance; sometimes it was also unclear whether an eye movement was due to an automatic salience effect or due to a controlled intentional strategy. As an example, think of the higher fixation rate on oncoming traffic [49,50] that could have reflected the attraction of the eyes by the salient movements of the oncoming cars or the controlled monitoring of an important characteristic of the current driving situation.

When we reviewed the eye movement findings of prior real-world night driving studies, we also noted that different subtasks seemed to be involved in driving: subtasks that could sensibly be distinguished from one another because performance on some of them was, for example, seemingly spared more by age than performance on other tasks. Therefore, we next propose a new description of the driving task in which we discriminate between three different groups of subtasks.

The first group of subtasks comprises the vehicle-related tasks. Their common denominator is that they all are primarily action-oriented and typically highly automatized [110]. These subtasks cover the operation of the gas and brake pedals, and the changing of gears. Experienced drivers almost completely automatize performance in vehicle-related tasks, like changing of gears [111]. It is sensible to distinguish these vehicle-related subtasks from other driving-associated tasks because visual perception is relatively indirectly related to the performance on the vehicle-related tasks. Therefore, the connection between eye movements and the performance on vehicle-related tasks could take different forms. First and foremost, the automatization of vehicle-related task performance could deliberate the eyes from monitoring performance in these tasks (i.e., of the feet or hand) and allow the usage of the eyes for the monitoring of the equally or more important road conditions.

In contrast to the vehicle-related tasks, road-related subtasks are guided by visual input in a relatively direct way. An example of a road-related sub-task is the distinct pattern of eye movements that is observed during the negotiation of right curves. To keep the lane, drivers fixate the right side of the road during right curves, which probably provides the necessary information to drive through the curve successfully [112]. Vehicle-related tasks, like steering, can show degrees of spatial compatibility between the motor control of effectors and the sensors, for example, when drivers gaze and steer in the same direction. Of further note, some road-related tasks, such as lane keeping, also seem to be spared by the loss of visual acuity in aged participants, possibly because performance in these subtasks does not require as much foveal vision.

Finally, during situation-related subtasks, the relevance of an object or a location for driving is determined by a more flexible interpretation of the situation. In general, experienced and inexperienced drivers show very different gaze patterns during driving, which probably reflects their different understanding of driving situations [86,91,113,114]. Another example is oncoming traffic at night. Oncoming traffic at night is not only a highly salient event (due to the headlamps of the oncoming traffic standing out against the dark surroundings), but it also triggers actions fitting to the situation, such as avoidance of looking into the headlights of the oncoming traffic or prompting the driver to manually switch from high beam to low beam to reduce glare-elicited drop in driving performance for drivers of the oncoming traffic (cf. [77]). Like in vehicle-related tasks, in situation-related tasks, the connection between particular eye movements and driving performance could be weak (e.g., to look away rather than at the headlights of oncoming traffic) but for a different reason than in vehicle-related tasks. Whereas in vehicle-related task performance automatization is the major reason why these tasks are not directly connected to particular eye movement patterns, in situation-related tasks the particular visual information would only be the basis for further, independent cognitive interpretations, akin to situation awareness à la Endsley [36].

Above, we have highlighted that future real-world night driving studies should best incorporate driving performance measures additionally to measuring eye movements. We believe that our description of the driving task through three different subtasks could be helpful in the context of such studies, as our task description would already provide theoretical reasons for expecting stronger links (e.g., between lane keeping and eye movements) or weaker links (e.g., between operating of the brake pedal and eye movements) between different variables. Thus, our task description could be helpful for formulating research hypotheses in the context of eye movements during driving. In particular, we believe that concepts such as automatization or compatibility, being deeply rooted in cognitive psychological research, could be helpful for integrating cognitive parameters into driving performance measurements.

## Conclusion

In this review, we summarized, interpreted, and evaluated the meaning of eye movement patterns found in real-world night driving studies against the background of existing descriptions of the task of driving. This was done to decide which factors are potentially harmful to driving performance at night. We concluded that much of the drivers' eye movements could have reflected sensible scanning strategies (i.e., for the boundaries of the field of safe travel). However, their eye movements also indicated that, compared to daytime driving, night driving was associated with a more restricted distribution of fixations. Whether this reflected a harmful effect on night driving performance is not entirely certain, but it remains a likely possibility. Finally, we concluded with a proposal for a more fine-grained description of the driving task that could help to bridge the gap between eye movements and other dependent variables in driving research. However, its application has to await future research, as most studies reviewed did not relate eye movement to other task performance measurements for the evaluation of real-world night driving performance.

## Declaration of Conflict

The authors declare that there is no conflict of interest regarding the publication of this paper.

## Acknowledgment

ZKW Group GmbH funded this research during their cooperation with the University of Vienna for the project “Nachtfahrpsychologische Aspekte der Wirkung von Hyperscheinwerfern”. We thank Peter Hartmann, Christian Büsel and Elena Throm for helpful discussions on this review, as well as two anonymous reviewers whose constructive feedback helped to improve this paper vastly.
